# MALDI-TOF Mass Spectrometry Revealed Significant Lipid Variations in Follicular Fluid and Somatic Follicular Cells but Not in Enclosed Oocytes between the Large Dominant and Small Subordinate Follicles in Bovine Ovary

**DOI:** 10.3390/ijms21186661

**Published:** 2020-09-11

**Authors:** Priscila Silvana Bertevello, Ana-Paula Teixeira-Gomes, Valerie Labas, Luiz Cordeiro, Marie-Claire Blache, Pascal Papillier, Galina Singina, Rustem Uzbekov, Virginie Maillard, Svetlana Uzbekova

**Affiliations:** 1 CNRS, IFCE, INRAE, Université de Tours, PRC, 37380 Nouzilly, France; pbertevello@gmail.com (P.S.B.); valerie.labas@inrae.fr (V.L.); lavcordeiro@gmail.com (L.C.); marie-claire.blache@inrae.fr (M.-C.B.); pascal.papillier@inrae.fr (P.P.); virginie.maillard@inrae.fr (V.M.); 2 INRAE, Université de Tours, ISP, 37380 Nouzilly, France; ana-paula.teixeira@inrae.fr; 3 CHU de Tours, INRAE, Université de Tours, PRC, CIRE, 37380 Nouzilly, France; 4 L.K. Ernst Institute of Animal Husbandry, Dubrovitzy 60, Podolsk, 142132 Moscow, Russia; g_singina@mail.ru; 5 Laboratoire Biologie Cellulaire et Microscopie Électronique, Faculté de Médecine, Université de Tours, 10, bd Tonnellé, 37032 Tours, France; rustem.uzbekov@univ-tours.fr

**Keywords:** ovarian follicles, lipid fingerprints, mass spectrometry imaging, oocyte, follicular fluid, follicular cells, extracellular vesicles

## Abstract

Lipid metabolism in ovarian follicular cells supports the preparation of an enclosed oocyte to ovulation. We aimed to compare lipid composition of a dominant large follicle (LF) and subordinated small follicles (SFs) within the same ovaries. Mass spectrometry imaging displayed the differences in the distribution of several lipid features between the different follicles. Comparison of lipid fingerprints between LF and SF by Matrix Assisted Laser Desorption/Ionisation Time-Of-Flight (MALDI-TOF) mass spectrometry revealed that in the oocytes, only 8 out of 468 detected lipids (1.7%) significantly changed their abundance (*p* < 0.05, fold change > 2). In contrast, follicular fluid (FF), granulosa, theca and cumulus cells demonstrated 55.5%, 14.9%, 5.3% and 9.8% of significantly varied features between LF and SF, respectively. In total, 25.2% of differential lipids were identified and indicated potential changes in membrane and signaling lipids. Tremendous changes in FF lipid composition were likely due to the stage specific secretions from somatic follicular cells that was in line with the differences observed from FF extracellular vesicles and gene expression of candidate genes in granulosa and theca cells between LF and SF. In addition, lipid storage in granulosa and theca cells varied in relation to follicular size and atresia. Differences in follicular cells lipid profiles between LF and SF may probably reflect follicle atresia degree and/or accumulation of appropriate lipids for post-ovulation processes as formation of corpus luteum. In contrast, the enclosed oocyte seems to be protected during final follicular growth, likely due in part to significant lipid transformations in surrounding cumulus cells. Therefore, the enclosed oocyte could likely keep lipid building blocks and energy resources to support further maturation and early embryo development.

## 1. Introduction

In mammals, an oocyte develops inside of ovarian follicle until an ovulation in tight communications with somatic follicular cells [1], notably surrounding cumulus cells (CCs), mural granulosa cells (GCs) and theca cell layers (THs). Along the follicular growth, antral cavity fills with follicular fluid (FF), coming from plasma and secretions from follicular cells. Within growing antral follicle, GC surrounding an oocyte differentiate into CC, and the enclosed oocyte substantially enlarges its size. Specialized stromal layers, the internal and external TH develop out of the follicular basal lamina and contains capillaries, fibroblasts, immune cells and specialized steroidogenic cells [2]. In growing antral follicles, GC and TH actively proliferate and secrete different factors, produce hormones and uptake large amounts of lipids and cholesterol from plasma [3]. During follicular growth, fatty acid (FA) metabolism plays fundamental role [4]. A final step of oocyte development before fertilization, oocyte maturation is especially sensible to FA oxidation in cumulus-oocyte complex (COC) [5], especially in bovine and porcine [6]; moreover, during oocyte maturation, lipid content significantly changed [7]. Lipids are the building blocks for cellular membranes and important energy source [8]. Lipids are also important actors in molecular signaling pathways, since some phospholipids are precursors to secondary messengers, and FAs are able to activate transcription factors [9]. Neutral lipids as triacylglycerols and steryl esters, serve as precursors to sex hormones; in addition, sphingolipids may control steroid hormone biosynthetic pathways [10]. Along the ovarian follicle growth, GC increase estradiol production by converting TH-derived androgens into estrogens [11] and become highly steroidogenic within the weeks leading up to ovulation [12]. Both GC and TH layers express specific genes involved in steroidogenesis and lipid metabolism [13], and transcriptomic patterns of GC and TH changed along final follicular growth in bovine [14,15,16]. In addition, analysis of lipids in bovine ovarian follicular cells and fluid using Matrix Assisted Laser Desorption/Ionisation Time-Of-Flight Mass spectrometry (MALDI MS) revealed very specific lipid fingerprints in GC, TH, CC, oocyte and FF [13]. These differences in lipid composition corroborate with specific expression patterns of lipid metabolism-related genes in somatic follicular cells [13,17] and oocyte–cumulus complex [18,19,20,21]. Each follicle therefore possesses molecular machinery to uptake and transform lipids to assure energy requirement, membrane synthesis and signaling to maintain follicular homeostasis and [13] and provide to oocyte the necessary building blocks for the first steps of embryo development [5,22].

In cycling ovary, the most of the follicles disappear through atresia; however, in each cycle a pool of antral small follicles (SFs) enter into the final follicular growth. In mono-ovulating species as bovine, one of such follicles becomes dominant in view of further ovulation. The size of ovarian follicles during final growth can reflect the capacity of enclosed oocytes to support meiotic maturation, fertilization and early embryo development. In bovine reproductive biotechnologies, the oocytes from the large potentially dominant follicles (LFs, >8 mm) are more competent for in vitro embryo development than the oocytes from the smaller ones (<6 mm) [23,24], although the oocytes themselves have similar size. However, accumulation of lipids in the oocytes correlates with their competence to in vitro embryo development [25,26]. In cows, lipid composition of COC environment affected oocyte quality, in vitro and in vivo [27,28,29]. FF of cows, which have received the diet supplementation with 1% dry matter of polyunsaturated FA *n*-3, was significantly enriched in *n*-3 PUFA, and correspondent enclosed oocytes have developed higher quality blastocysts compared to control animals [29]. In addition, supplementation of 1 μM of *n*-3 PUFA docosahexaenoic acid during in vitro maturation of bovine oocytes resulted in their higher blastocyst rate after in vitro fertilization [27]. Moreover, lipid composition of PUFA *n*-3 treated oocytes in vivo and in vitro significantly differed compared to control ones [28,29].

Role of lipid metabolism in the follicular dominance is not enough known. Follicle final growth is accompanied by significant changes in follicular cells metabolism and FF composition. Concentrations of different ions, total proteins, glucose, FAs and other metabolites in FF significantly varied from the small to large follicles in cows [30]. Transcriptomic analysis performed on bovine GC and internal TH from the small (subordinate) and large (dominant) follicles showed significant differences in expression of different genes, including those involved in FA metabolism and related to increased steroidogenic capacity of the larger follicles [31,32].

The objective of this study was to compare lipid content of FF, somatic follicular cells and enclosed oocytes between the LF and SF within the same bovine ovaries, in order to enlighten differences in the follicular environment of the oocytes with different potential to ovulate.

## 2. Results

### 2.1. Analysis of Lipids by Mass Spectrometry Imaging (MSI) on Bovine Ovarian Sections

**Figure 1:**
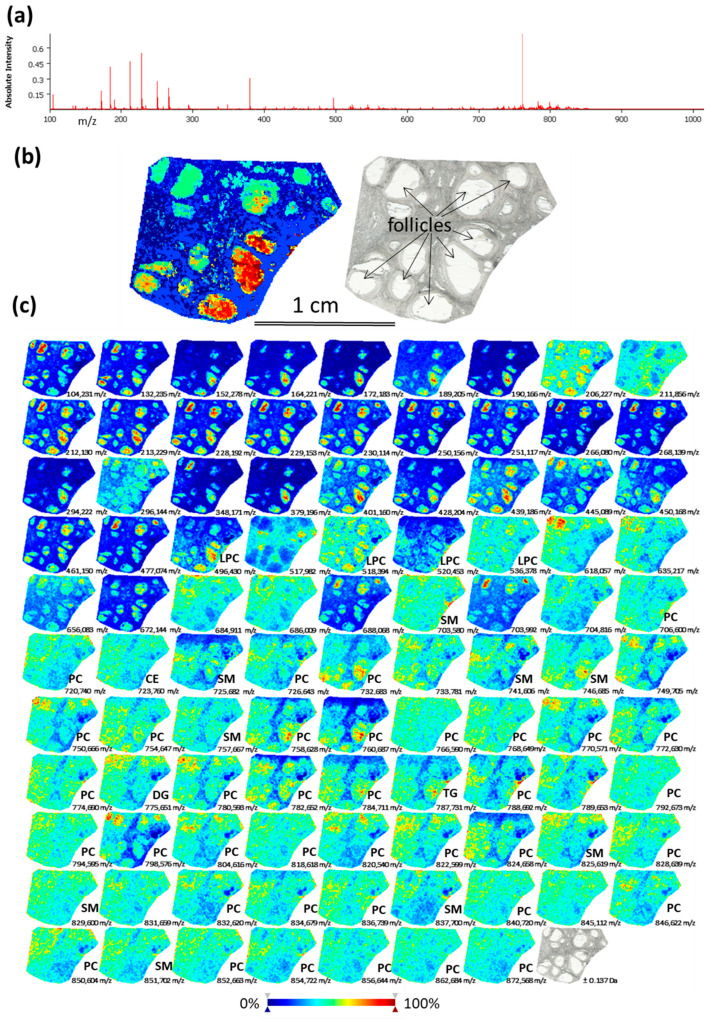
Detection of lipid species in bovine ovary using Matrix Assisted Laser Desorption/Ionisation Time-Of-Flight (MALDI-TOF) mass spectrometry imaging (MSI). (**a**) Skyline projection spectrum of 281 molecular species in the 100–900 *m*/*z* range. (**b**) Spatial segmentation map (left picture) was obtained by hierarchical clustering of lipid profiles using the bisecting k-means algorithm. Light scan of the same section (right picture). (**c**) Images of ion density maps of detected *m*/*z* species through ovarian section. Lipid classes of 53 identified features are shown (see Supplementary data Table S2 for exact annotations). CE—cholesteryl ester; DG—diacylglycerol; PC—phosphatidylcholine; LPC—lysophosphatidylcholine, SM—sphingomyelin, TG—triacylglycerol.

MSI was performed on bovine ovarian cryosections in order to observe spatial distribution of lipids through ovarian compartments. The skyline projection spectra were generated from all the detected ion signals (Figure 1a). MSI allowed the mapping of 281 molecular species in the mass range 100–900 *m*/*z* with variable intensity within the regions of interest (Supplementary data, Table S1). All the ion spectra were grouped according to their similarity using unsupervised hierarchical cluster analysis. The most specific clusters were selected to generate representative molecular reconstruction of ovarian tissue sections (MSI segmentation map, Figure 1b, left picture). Follicles and interstitial stroma compartments are distinguished in coherence with histological digital image of the same section (Figure 1b, right picture). The different colors in MSI segmentation map represent the differences in lipid composition between the different zones throughout ovarian sections, and between the different follicles. Ion density maps for the mostly abundant lipid features are presented in Figure 1c: different colors in each ion density map represent different abundance of this lipid in situ (color bar: increasing from blue to red).

Annotation of 53 lipids (18.8% of all detected *m*/*z* features) was performed as described earlier [13]. Identified lipid species were either protonated ([M + H]^+^) or contained salt adducts ([M + Na]^+^ or [M + K]^+^). Identified lipids represented the following classes: phosphatidylcholines (PC)—55.6%; lyso-phosphatidylcholine (LPC)—7.4%; sphingomyelin (SM)—16.7%; ceramide (Cer)—1.9%; cholesteryl esters (CE)—5.6%; diacylglycerol (DG)—1.9%; triacylglycerol (TG)—9.3% (Supplementary Table S2). According to ion density maps, several PC (*m*/*z* 766.59; *m*/*z* 774.66, *m*/*z* 840.72), SM (*m*/*z* 703.58, *m*/*z* 829.60, *m*/*z* 851.70) and CE (*m*/*z* 723.76) were heterogeneously distributed through the ovarian sections, including interstitial stroma and the follicles (Figure 1c). Several LPC as *m*/*z* 496.43 (LPC 16:0); *m*/*z* 518.39 (LPC 18:3); *m*/*z* 520.45 (LPC 18:2) were more abundant inside the follicles and preferentially located to FF. Several PC, such as *m*/*z* 758.628 (PC 34:2/), *m*/*z* 760.87 (PC 34:1), *m*/*z* 824.658 (PC 36:2) showed similar pattern. While other lipid species showed more diffuse and heterogeneous location throughout the ovary, as *m*/*z* 703.580 (SM 43:1), *m*/*z* 732.683 (PC 32:1), *m*/*z* 851.702 (SM 43:1) and 872.568 (PC 40:6). Globally, using MSI, the follicles were clearly discriminated by their lipid content from the ovarian stroma tissues. Moreover, MSI revealed that lipid content in FF of different follicles might differ.

### 2.2. Comparison of Lipid Profiles in Follicular Cells and Fluids of Small and Large Follicles

**Figure 2:**
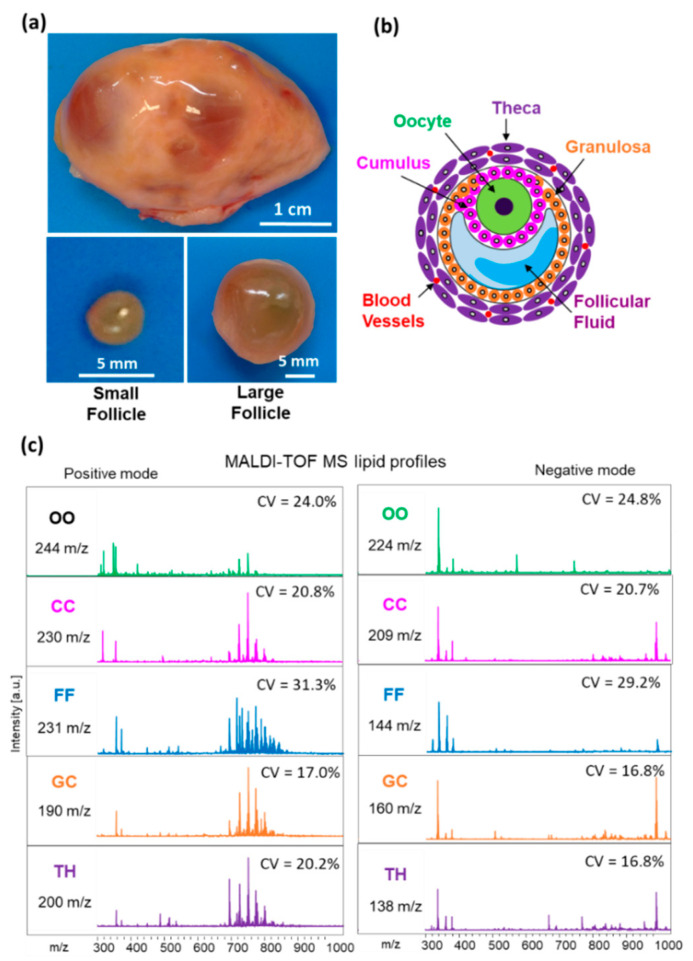
MALDI-TOF MS lipid profiling of follicular compartments. (**a**) Representative image of bovine ovary and dissected small (*n* = 12) and large (*n* = 12) follicles. (**b**) Schematic representation of ovarian follicle with cellular and fluid compartments. (**c**) Representative MALDI-TOF MS spectra of bovine individual oocytes (OO), cumulus cells (CC), granulosa (GC), theca cells (TH) and follicular fluid (FF) in positive and negative acquisition modes, *m*/*z* range of 300 to 1000. Number of features (*m*/*z*) detected and mean coefficient of variation of their abundance (CV%) are indicated for each compartment and acquisition mode.

In order to compare lipid composition between the SF and LF, we have analyzed MALDI MS lipid profiles of GC, TH, CC, oocyte and FF in the groups of follicles of different size. Lipid fingerprints obtained from both positive and negative acquisition modes, in each follicular compartment, contained in total 338, 350, 375, 439 and 468 peaks detected in TH, GC, FF, CC and oocytes, respectively (Figure 2). For all these detected features, coefficient of variation (CV%) was generally lower than 25% in all the follicular compartments, except FF profiles where the mean CV% was 30.3%.

The number of differently abundant features between SF (*n* = 12) and LF (*n* = 12) significantly varied regarding different compartments (Table 1). In oocytes and TH, only 1.7% and 5.3% features, respectively, significantly varied between the LF and SF (*p* < 0.05, fold change >2). In CC and GG, the percentage of differential species was 9.9% and 14.9%, respectively. At the same time, in the FF more than half of peaks (55.5%) significantly changed their abundance in LF as compare to SF.

Table 2, Table 3, Table 4, Table 5 and Table 6 present annotated differentially abundant lipids between the SF and LF, in FF, oocytes, CC, GC and TH, respectively. Data on all the peaks detected in these compartments and possible annotations are reported in the supplementary data (Table S3 and Table S4, respectively). Lipid identification was based by the lowest delta value. Adducts are marked in the brackets. [H]^−^- ion in negative mode, [H]^+^- ion in positive mode. Phospholipids with ‘O-’ prefix is used to indicate the presence of an alkyl ether, whereas the ‘P-’ prefix is used for the alkenyl ether (or Plasmalogen) respectively.

In FF, among the 49 annotated differential lipids (Table 2), mainly PC and LPC were more abundant in large follicles (9 PC, 4 LPC, 2 CE, 1 LPE or LPC, and 1 SM), whereas DG, SM, PE and PI were over-represented in the smaller follicles (12 PC, 8 PE, 3 PI, 3 DG, 2 SM, 3 PS and 1 LPC). In addition, the incidence of lipids including polyunsaturated FAs in FF was higher in SF (35%) than in the large ones (20%)—as much as 15% saturated FAs and 17% monounsaturated FAs in SF.

In the oocytes, two PC and one SM features were more abundant in the smaller follicles (Table 3).

In CC, among 43 differential lipid species, 15 PC, 2 SM and 1 TG significantly more abundant in LF compared to SF were identified; 44% of these lipids contain very long chain polyunsaturated carbons (Table 4).

In GC, among the 35 differential features that were more abundant in LF, only one was annotated as PI 36: 2 (Table 5). Among the lipids, over-represented in the SF, we annotated C18:0 carnitine, 3 LPC, 2 PC, 1 SM and 1 PE. 55% of these lipids showed the presence of medium or long-chain polyunsaturated FAs (Table 6).

In TH, four peaks that were more abundant in LF, corresponded to three identified lipids: (DG 38:4) and PE (34:2 and 38:7) (Table 6).

**Figure 3:**
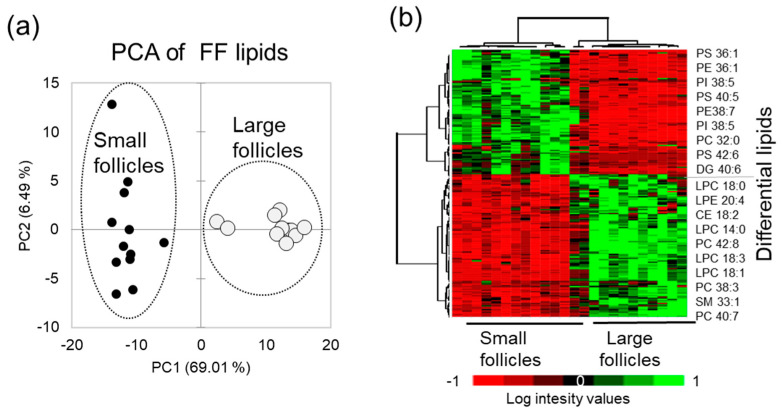
Comparative analysis of follicular fluid from the small (*n* = 12) and large (*n* = 12) follicles. (**a**) Principal Component Analysis (PCA) shows the difference between the small and large follicles MALDI PS lipid profiles. (**b**) Relative abundance of differential lipids in FF presented as a heatmap; the most differential annotated lipid features are shown at the right side of the heat map.

Principal Component Analysis (PCA) performed using relative abundance values of the differential lipid features demonstrated that FF lipid profiles clearly discriminated SF and LF groups (Figure 3a). Relative abundance of 111 up-regulated and 97 down-regulated differential lipid species in FF from the LF and SF are here presented as a heat map, and the most differentially abundant identified lipids are indicated on the right side of the heat map (Figure 3b).

**Figure 4:**
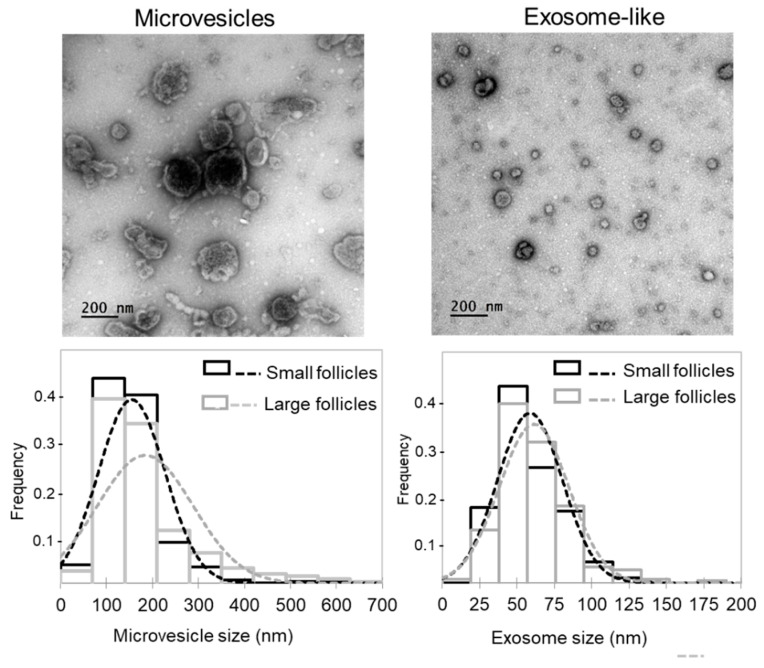
Analysis of extracellular vesicles (EVs) extracted from follicular fluids of small and large bovine ovarian follicles. Representative electron microscopy microphotographs of microvesicles (MVs; pellets after 12,000× *g* centrifugation) and exosome-like EVs (pellets after 100,000× *g* centrifugation) are shown. Histograms present comparison of size frequency distribution of microvesicles and exosome-like EVs between the SF and LF groups. In total, 612 MVs and 608 exosome-like vesicles were taken for analysis.

Whereas extracellular vesicles present in FF (ff-EVs) likely contribute to FF lipid profiles, we extracted ff-EVs from a number of SF and LF, and performed comparative analysis. We evidenced that our ff-EV preparations mostly contained either microvesicles (MVs; pelleted at 12,000 g, vesicle diameter more than 100 nm) or exosome-like nanovesicles (pelleted at 100,000 g, diameter 30–100 nm) (Figure 4), as defined in minimal information for studies of extracellular vesicles (MISEV) [33]. As shown, distribution of EV sizes in the fractions containing the MVs was different between the LF and SF, whereas exosome-like fractions from LF and SF were similar. Moreover, the mean diameter of MVs in LF (184.1 ± 5.9 nm, *n* = 316) and SF (155.5 ± 4.0 nm, *n* = 321) significantly differed (*p* = 0.00006). In contrast, mean exosome diameter from SF and LF was not different (*p* = 0.164): 61.4 ± 0.08 nm (*n* = 304) and 58.9 ± 0.07 nm (*n* = 312), respectively. However, protein concentration in exosome-like preparations from large dominant follicles was significantly lower (*p* < 0.05) than from smaller subordinated ones (23.5 ± 3.0 μg/mL and 64.5 ± 4.8 μg/mL, respectively, *n* = 6 per group).

**Figure 5:**
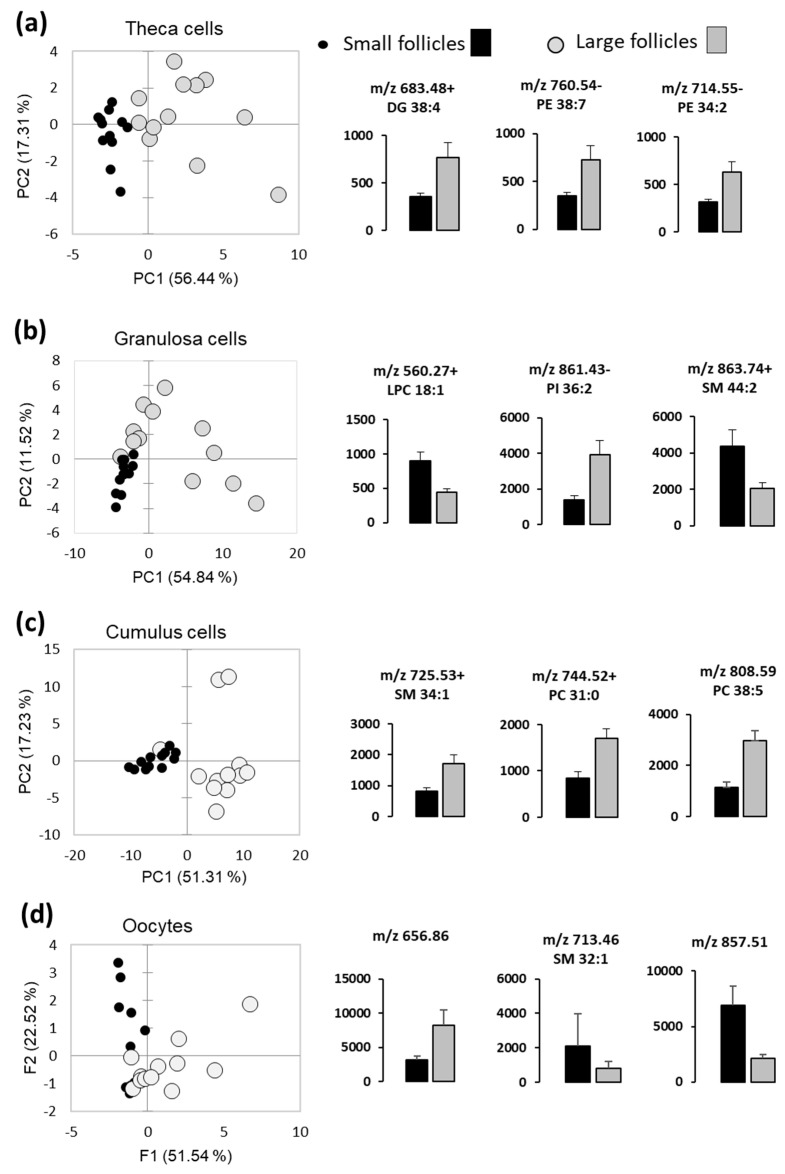
Differential analysis of MALDI-MS profiles of follicular cells and fluids in small and large follicles (*n* = 12 per group). Principal Component Analysis (PCA) shows the difference between the small- and large-follicle lipid profiles in theca cells (**a**), granulosa cells (**b**), cumulus cells (**c**) and in the oocytes (**d**). Histograms present relative abundance values (mean of 12 independent samples ± SEM) of significantly different lipid features (*p* < 0.05).

The differences of lipid fingerprints of follicular somatic cells and the oocytes between the LF and SF were analyzed using PCA, which was performed with relative abundance values of differential lipids in these compartments. Globally, PCAs were able to discriminate LF and SF, however some overlay between the LF and SF groups were observed (Figure 5). Examples of the lipids differentially abundant between the LF and SF (*p* < 0.05) are presented for theca cells (Figure 5a), GC (Figure 5b), CC (Figure 5c) and oocytes (Figure 5d). Relative abundance values of all detected lipids are presented in Supplementary data, Table S3.

### 2.3. Gene Expression Analysis

Expression of candidate genes involved in transport of lipids (APOA1, CD36, CPT2, FABP3, FABP5, SCARB1, SCARB2), FA metabolism (ACACA, ACADVL, ACOT9, HADHA, LPL, PLIN2, TRIB2), lipid antioxidant peroxidase (GPX4), steroidogenesis (CYP11A1 and HSD3B1) and glucose transport (GLUT1), were here compared in TH and GC between the SF and LF (*n* = 12 per group) by real-time PCR (Table 7). Relative expression of HSD3B1 was twice lower in TH layer and CYP11A1 6-fold higher in GC of LF as compared to small ones (*p* < 0.05). In TH, genes ACOT9, PLIN2 and TRIB2 were significantly higher expressed in SF than in LF (*p* < 0.05). Genes CD36, CPT2, FABP3, FABP5 and LPL tended to overexpression in theca of SF (*p* < 0.1). In GC, relative expression of GPX4 was significantly higher (*p* < 0.05) and that of ACADVL, HADHA tended to be higher in LF as compared to SF (*p* < 0.1).

### 2.4. Quantification of Neutral Lipids in Ovarian Follicles of Different Size

**Figure 6:**
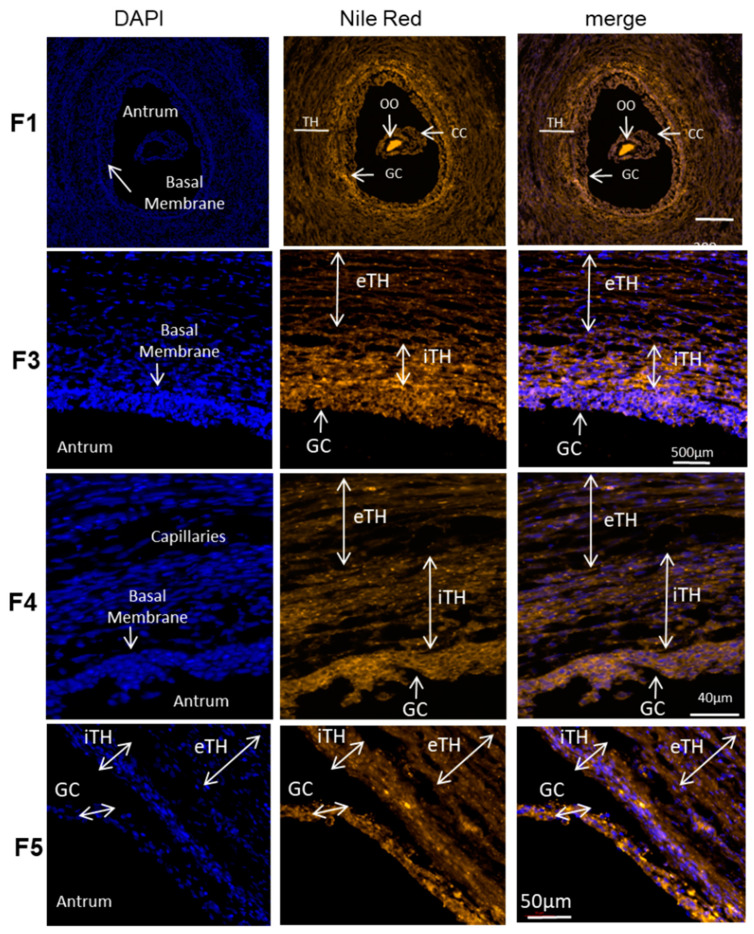
Total lipid detection with Nile red fluorescence in ovary follicular compartments. Representative images of Nile red (neutral lipids and phospholipids) and DAPI (nucleus) staining in bovine follicles of different size (F1, F3, F4, F5). The mean size of the follicles per group was 0.88 ± 0.08 mm for F1 group; 4.05 ± 0.12 mm in F3 group; 6.06 ± 0.22 mm in F4; 12.00 ± 0.82 mm for group F5. iTH—internal theca, eTH—external theca layer, GC—granulosa cells, OO—oocyte.

Spatial distribution of total lipids in the follicles of different sizes was observed on histological sections of the ovaries treated with Nile Red, lipophilic agent that stains intracellular lipid droplets (here in orange) (Figure 6). The oocyte demonstrated the strongest labeling, and, according to fluorescence intensity, the concentration of lipid droplets seems differ between the GC and TH cell layers in the follicles of different size. The follicles were divided to five groups according to their size. The mean size of the follicle group F1 was 0.88 ± 0.08 mm (*n* = 6), group F2 (*n* = 6)—2.27 ± 0.12 mm; group F3—4.05 ± 0.12 mm (*n* = 11); group F4—6.06 ± 0.22 mm (*n* = 12) and group F5—12.00 ± 0.82 mm (*n* = 8).

**Figure 7:**
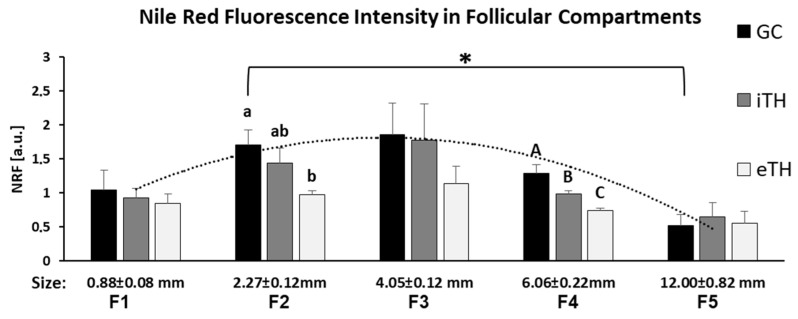
Graphical representation of mean Nile Red fluorescence (NRF) intensity distribution in granulosa (GC), internal theca (iTH) and external theca (eTH) layers in five groups of ovarian follicles of different size (F1-F5). Histogram bars are mean NRF values ± SEM. Different letters mean significant difference (*p* < 0.05) between the follicular layers inside each groups. * denotes significant difference of NRF in GC between the small and large follicles (F2 and F5 group, respectively).

Nile Red Fluorescence (NRF) was quantified in internal and external theca layers (iTH and eTH, respectively), GC and FF of the follicles of different size from F1 to F5, and compared between the groups (Figure 7). The highest NRF levels were observed in somatic cells of the small follicles, particularly from F2 and F3 groups, compared to other follicular size groups (Figure 7). GC and TH cells of small F2 follicles showed three-fold higher NRF levels than in large F5 follicles (*p* < 0.05). Globally, no significant difference between GC and TH layers was observed, except for F2 and F4 groups, where GC showed higher NRF level compared to eTH (F2) or to both theca layers (F4). In F4 group, NRF in iTH was significantly higher than in eTH (*p* < 0.05).

### 2.5. Caspase-3 and Lipid Accumulation in Bovine Follicular Cells

**Figure 8:**
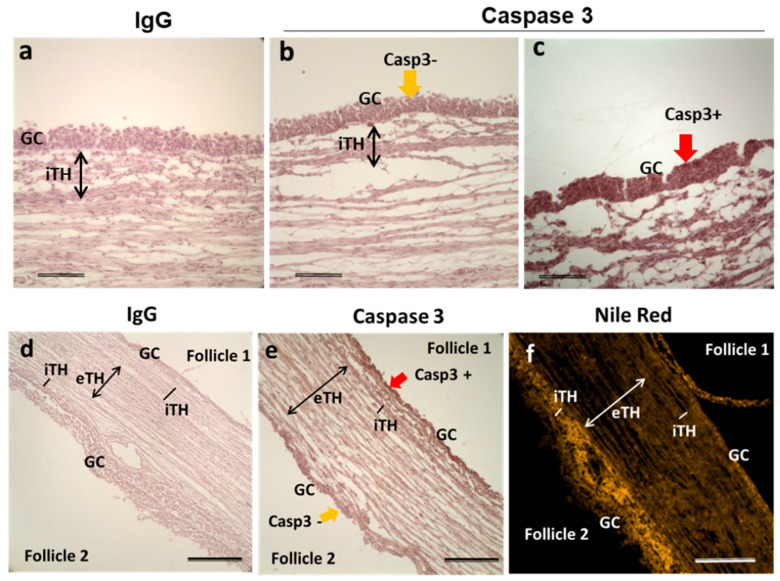
Representative image of immunohistochemistry to detect total caspase-3 in ovarian follicles, here group F4 (6.06 ± 0.22 mm). Labeling with rabbit IgG instead of caspase-3 antibody (**a**,**d**), with caspase-3 antibody (**b**,**c**,**e**) and staining with Nile Red fluorescence (**f**). Caspase-3 labeling was observed in GC of potentially atretic follicles (**c**,**e**—follicle 1). GC with no positive Caspase-3 labeling (**b**,**e**—follicle 2). Bars 200 μm (**a**–**c**) and 400 μm (**d**–**f**). Abbreviations: Casp3—caspase-3, GC—granulosa cells, iTH—internal theca cells, eTH—external theca layer, yellow arrow—caspase-3 negative labeling; red arrow—caspase-3 positive labeling.

In the ovary, among the follicles of the same size there are growing and atretic ones; the first may become dominant whereas the second will die through atresia. We would like to observe lipid accumulation in follicular cells of atretic and healthy follicles. In order to differentiate the follicles in advanced atresia from those in final growth, immunostaining against caspase 3, a protein involved in apoptosis cascade and used as an indicator of atresia in mouse [34] and human [35] ovaries, was performed. Adjacent ovarian sections to those, which were tested by NRF, were stained with anti-caspase-3 antibody. Morphology of GC nuclei coupled with Casp3 immunohistochemistry allowed estimation of follicular atresia degree (Figure 8). Highly atretic follicles presented mostly picnotic nuclei and strong Caspase-3 labelling in GC (Casp3+; Figure 8c,) whereas more healthy follicles showed faint Caspase-3 labeling in GC (Casp3-; Figure 8b), similar to IgC control staining. In all three ovaries analyzed, Nile Red staining appeared less pronounced in GC of Casp3- antral follicles (*n* = 5) comparing to Casp3+ follicles (*n* = 7), but tendency to lipid accumulation was inversed in internal theca (iTH) layer (Figure 8f). Figure 8e represented the example of two neighboring follicles with different degree of atresia: follicle 1 was highly atretic (Casp3+) and whereas follicle 2 was not (Casp3−).

## 3. Discussion

### 3.1. MSI Mapping of Lipids Within Ovarian Section Revealed Difference Between the Antral Follicles

In the present work, lipid mapping performed on bovine ovary sections by MS imaging displays spatial distribution of a range of different features within the ovarian follicles and interstitial tissues similarly to previously reported MSI of lipids in porcine and mice ovaries [36].

As expected, MSI clearly distinguished antral follicles from ovarian stroma, and FF inside the follicles within the same ovary presented different lipid composition. Interestingly, our study revealed LPC 16:0, PC 32:1; PC 34:1, PC 37:3 and a number of non-annotated low weight lipid species, which were differentially abundant between the follicles. Among them, LPC 16:0 and PC 34:1 were significantly higher abundant in FF of smaller follicles as was demonstrated by MALDI MS profiling. Recent MSI study demonstrated that ovarian stroma, corpus luteum and epithelium of follicular wall have similar lipid signatures in mice, porcine and bovine, and the major differences were observed in steroidogenic structures as corpus luteum [37]. In addition, MSI on mice ovarian sections revealed significant increase in the intensity of various lipids in post-ovulated ovaries comparing to follicular stage [38]. In our work, bovine ovaries without apparent corpus luteum were analyzed by MSI, and the differences between the follicles were observed. Ovarian follicles are very dynamic structures, and steroid composition in follicular fluid varied along the follicle growth and massive proliferation of GC [39]. Moreover, significant increase of high-density lipoproteins during follicular growth and maturation was reported in cows [40]. In addition, antral follicles may be at different stage of atresia, and that could affect FF composition due to the modulation of different GC secretions and growing abundance of released apoptotic bodies. Summarized, these processes likely contributed to the differences in lipid composition between the follicles, revealed by MSI.

### 3.2. Lipid Fingerprints Changes along Follicle Growth Are Specific to Follicular Compartment

In the present study, we have compared lipid profiles of large potentially dominant follicles and small subordinated follicles within the same ovaries and observed the variations in lipid abundance in their follicular cells. However, the most significant difference between LF and SF was observed in FF lipids. Indeed, in bovines, the healthy small follicles (<8 mm) are usually androgen-dominated, whereas the large follicles (>11 mm) are estrogen-dominated. This is in line with differential expression of steroidogenic enzymes between the follicles of different size that we here observed in GC and TH cells. Thus, *CYP11A*, involved in estradiol production, was higher expressed in GC from LF compared to SF; whereas HSD3B1, involved in progesterone production, was higher expressed in TH from SF compared to LF, confirming the endocrine characteristics of follicular cells in different growth phases. We demonstrated that intracellular and FF lipids changed along with follicular growth. Indeed, FF is an environment for oocyte growth and maturation; therefore, FF composition reflects follicle metabolic state, which is primordial for the competence of enclosed oocyte. Variations of lipid profiles between the large dominant and small subordinated follicles revealed be cell specific. Enclosed oocytes demonstrated only few changes whereas more than half of the lipids varied in FF. Identified differential lipids may indicated significant changes of membrane composition in both follicular cells and EVs and eventual potential changes in the relative abundance of signaling lipids and lipoproteins within these compartments, during follicle growth.

### 3.3. Follicular Fluid Lipid Composition is the Most Different Compartment between the Large and Small Follicles

The most significant variations in lipid composition between large and small follicles were observed in FF. Although FF reflected metabolic composition of blood serum, the concentration of different metabolites, FAs and steroid hormones significantly differed between these fluids [31]. FF contain floating cell remnants and numerous membrane-coated EVs that represented by apoptotic bodies, microvesicles and exosomes [41], which cargo lipids of different classes. So significant differences of lipid composition in cell-free FF between the SF and LF are likely due to content of released EVs. The EVs are enriched in cholesterol, SM, glycosphingolipids and phosphatidylserines that have a structural role in exosome membranes [42]. Such lipids species were found in among the differential lipids between LF and SF fluids. Moreover, EVs concentration was significantly lower in FF of the larger follicles compared to SF in cows [43], in line with our observations. Interestingly, lipid content in the exosomes demonstrated different enrichment factor from the originating secreting cells [42]. Therefore, the differences in FF lipid composition between the follicles of different sizes could be explained, at least in part, by releasing of different EVs by corresponding follicular cells. Indeed, GC have different proliferative and secretion activity in the follicles at different stages, and it was confirmed by comparative analysis of corresponding transcriptomes [15]. In the present study, we demonstrated that FF microvesicles in LF were significantly larger than in SF, and exosome-like vesicle concentration was lower in LF that in SF, as was also demonstrated earlier [43]. Thus, differences in EVs may contribute to enormous differences of lipid abundance in FF of dominant and subordinate follicles; however, more detail studies must be performed on purified EVs and EV-free fluid in order to evaluate a part of EVs in FF lipid fingerprints.

In our study, among the identified lipids, 14–18 carbon saturated or monosaturated LPC species (LPC 14:0, LPC 16:0, LPC 18:0, LPC 18:1, LPC 18:3) and cholesterol esters CE 18:2 and CE 18:3 were more abundant in large dominant follicles compared to SF. Over-abundance of some specific lipids, including LPC in FF might determine the quality of the enclosed oocyte in human [44]. Interestingly, lower LPC concentrations in human adipocytes were associated with some pathologies, as an obesity [45], which affects fertility. LPCs are associated to activate several second messengers, including extracellular-signal-regulated kinases and protein kinase C [46], which are involved in the regulation of follicular development and oocyte maturation. LPCs serve as “eat-me” signal released from apoptotic cells due to caspase 3 mediated activation of phospholipase A2 [47] and thus involved in inflammation and apoptotic cell death—the processes intensifying during final stages of follicular growth. Thus, higher abundance of these lipids in FF corroborates with more apoptotic follicular environment. Indeed, follicular GC layer in LF contains more apoptotic cells than smaller ones [48]. Interestingly, in bovine oocytes after *n*-3 PUFA supplementation in vitro or in vivo, LPC 16:0 and/or LPC 18:0 were more abundant in lower quality oocytes [28,29]. In addition, different lysophosholipids derived from EVs or from lipoproteins [49] participate in intra-follicular signaling and cell-to-cell communication [50]. These processes have different dynamics through follicle growth and might in part determinate the follicle destiny—to go or not to go to atresia.

Cholesterol esters are also the components of lipoproteins, and thus higher abundance of CEs in larger follicles might be related to lipoprotein concentrations in FF. We confirmed that apolipoprotein A1 gene expressed in both GC, TH cells and in CC [13]. ApoA1 is the main protein associated with HDL, and the HDLs are the most represented lipoproteins in FF, moreover HDL concentration increased with follicular final growth [40]. In fact, HDLs supply cholesterol for steroidogenesis [51], thus total cholesterol increase could be explained by a temporary halt in steroid synthesis by the follicles before becoming dominant or atretic [52]. HDLs also participate in protecting the cells against oxidative stress. Strong expression of glutathione peroxidase 4 (*GPX4*) in both GC and TH showed the importance of this antioxidant enzyme, which has the capacity to scavenge free radicals, prevent lipid peroxidation and maintains intracellular homeostasis as well as redox balance [53], important for preparation of ovulation.

Phosphatidylcholines PI 38:3, PI 38:4 and PI 38:5 were 3–10-fold more abundant in FF of subordinated follicles compared to LF. Intriguingly, in mice ovary, the PI 38:4 was mostly present in *corpus luteum*, a steroidogenic structure, which replaces the follicles after ovulation and uses precursor lipid species for steroid production [38]; therefore these abundant PIs may serve for increasing requirement of steroid production during follicular growth.

Interestingly, difference in lipid composition between the follicles of different size was much more significant in FF than in GC. Thus, enzymatic activity transforming lipids in FF likely may also play a role [54]. In fact, different enzymatic activities related to FA metabolism were shown in cell-free FF: lysophospholipase D [55], paraoxonase 1 (PON1) [56], estradiol esterification [57], catalase, glutathione peroxidase, glutathione reductase and superoxide dismutase (SOD) [58] activities in women, and SOD [59] and PON1 [60] activities in bovine FF.

### 3.4. Lipid Changes in Granulosa Cells During Follicle Growth Impact Follicular Fluid Composition

During follicular growth, GC layers change proliferation activity and increased secretion of steroid hormones especially close to ovulation [61]. In GC, one of the upregulated lipids in LF was annotated as PI 36:2, whereas PC, LPC, SM, PE classes and carnitine were more abundant in subordinated SF. Comparative proteomic study performed on GC from LF and SF, reported different levels of eight proteins involved in PI3K-signaling [62]. Moreover, significant upregulation of proteins involved in steroid biosynthesis and fertilization was observed in LF, whereas proteins more abundant in SF were associated to cell communication and signal transduction [62]. These data corroborate with differential abundance of PI 36:2 and other lipid species between these LF and SF in our study. Indeed, PIs are the minor acid phospholipids present in cell membranes, which play a major role in cell interactions and regulate the activity of integral membrane through phospholipase-mediated releasing of DG and phosphorylated inositols (PIPs)—powerful second messengers [63]. These PIPs are crucial for regulation of follicular growth and oocyte maturation [64]. Observed variation of PI 36:2 was in line with the upregulation of phospholipase C and D genes in bovine GC of LF compared to SF [65].

In addition, three LPC (16:0 and 18:1 and 20:4) were 2.5-fold less abundant in GC from LF compared to SF, and this LPC down-regulation was observed in only GC, but neither in CC nor TH cells. Interestingly, LPC 16:0 was also less abundant in the oocytes of higher quality, after *n*-3 PUFA supplementation *in vitro* or *in vivo* [28,29], suggesting that this lipid might be associated with higher developmental potential of the oocyte. Regarding the genes encoding the apolipoproteins, *APOA1*, *APOA2* and *APOE1* were also upregulated in granulosa of LF [31]. Taken together, significant differences in GC lipid composition reflect functional differences between the growing dominant and regressing SF, that are likely related to cell proliferation, steroidogenesis, different enzymatic activities, FA transformation, protein transport, matrix synthesis and signaling, as reported by analyses of GC transcriptomes [31].

### 3.5. Minor Lipid Changes in the Oocyte Contrasted to Lipid Modulations in Surrounding Cumulus Cells During Follicle Growth

Differences in relative abundance of lipids in the oocytes from SF and LF concerned only 8 out of 468 detected features, among them we identified PC 29:1 and SM 32:1. Our data corroborate with a recent study showing little variations in seven phospholipids, from PC and SM classes between the oocytes from less than 2 mm to more than 8 mm bovine follicles [66]. In large dominant follicle oocytes, structural changes as Golgi complexes size decreased, peripheral location of cortical granules and increase in lipid deposit were observed in contrast to smaller subordinate ones [67]; moreover, enrichment and translocation of mitochondria were also evidenced [52]. Contrarily, very faint fluctuations in phospholipid profiles between the oocytes from LF and SF may suggest that during terminal stages of follicular growth, membrane integrity of immature oocyte seems be highly protected from changing FF lipid environment by surrounding cumulus cells.

CC express necessary enzymes to metabolize the huge amounts of FAs and cholesterol for primary production of steroids and transform lipids to provide energy or stock lipid reserves [20,21,52]. In contrast to enclosed oocytes, about 10% of lipids in CC showed differential abundance between dominant LF and subordinate SF, and 95.3% of differential lipids were more abundant in LF. Among them, we identified mainly PC and SM that may indicate the profound changes in membrane components of CC. Sphingolipids are involved not only in the stabilization of membrane structure, but also in cell signaling and cell–cell recognition [68]. This signaling system is evolutionarily conserved, analogous to conventional systems, such as the cAMP and PI-pathways. The SM pathway uses ceramide (generated from SM) as a second messenger. Ceramide signaling is a stress response system and transduces signals mediating differentiation, growth, cytokine biosynthesis and secretion, and a variety of other cellular functions [69]. As any cells, CC secrete EVs, which are enriched in specific lipids, notably SM. Thus, EVs emanated from prostate cancer cells were 2–4 times enriched in sphingosine-containing lipids as ceramide, SM and gangliosides, in contrast to PCs more enriched to the cells [42].

Significant 2.8-fold increase in TG (47:1) and numerous PCs in CC of LF may indicate more lipid inclusions in CC in dominant follicles that corroborates with an increase in intracellular lipid droplets in CC during *in vitro* maturation of COCs [21]. Inhibition of FA oxidation decreased accumulation of lipid droplets in CC and led to their death [21]. CC play essential role in protection of the enclosed oocyte from lipotoxicity through lipid droplets accumulation within CC cytoplasm [70], notably by transforming saturated FAs through SCD (stearoyl-CoA desaturase) activity [20,71].

It was shown, that oocyte competence to support *in vitro* embryo development varied in relation to follicular size [72,73] and also to apoptosis degree in CC [74,75]. In fact, during *in vitro* maturation, apoptotic rate in CC increased [76], lipid profiles of CC significantly changed and expression of the genes of FA oxidation intensified [21]. Therefore, significant variations of lipid composition in CC of dominant follicles may activate the mechanisms of protection of enclosed oocyte. In fact, only the oocytes from the follicles in the regressing phases had more lipid volume compared to oocytes from the follicles during terminal growth, maturation and ovulation [52]. Differences in CC lipids between the SF and LF reflects different lipid composition of surrounding FF and FA transformation activity in these cells [21,77]. Besides, similar differential lipids in FF and CC (PC32:0, PC34:0, PC 36:1, PC 38:4, PC 40:6), probably indicate that CC may enrich FF lipidome through EVs secreted by these cells.

### 3.6. Lipid Changes in Theca Cells

TH layer is transpierced by blood capillaries, so circulating lipid species were also detected in MALDI MS profiling. Indeed, only 18 out of 338 features showed differential abundance between LF and SF. This mitigated difference corroborates with relatively stable transcriptomic profile of internal TH layers during antral follicle growth [32], unlike that of GC [31] where very numerous genes were differentially regulated between the SF and LF. However, we found over-expression of some genes related to lipolysis and FA oxidation in thecal cells of SF (*ACOT9, PLIN2, LP*L and *TRIB2*). Thus, higher fatty acid oxidation (FAO) activity, associated to higher lipid uptake from plasma, may support particular importance of these processes during follicular growth.

Mitochondrial FA beta-oxidation of fatty acyl-CoA is the major route of FA degradation, but very-long-chain FAs and branched-chain FAs are poorly oxidized in mitochondria and easily in peroxisomes [78]. Thus, numerous acyl-CoA thioesterases (ACOTs) reverse this reaction, despite the high-energy cost of acyl-CoA synthesis. ACOTs promote acyl-CoA hydrolysis, to provide the very high requirement free coenzyme (CoA), a cofactor of mitochondrial FAO. We demonstrated that *ACOT9* significantly overexpressed in SF and was more abundant in TH compared to GC. In somatic cells, ACOT9 operates to ensure sufficient free CoA to maintain optimal mitochondrial function [79] and participates in hydrolysis of medium and long-chain acyl-CoAs and CoA esters [80] that may have a role also in follicular growth and differentiation.

### 3.7. Lipid Accumulation in Follicular Cells Associated with Apoptotic State of the Follicles

Except the oocytes, which present a high amount of lipid inclusions, the follicles accumulate some lipid droplets mostly in GC and internal TH layers, as shown by Nile red staining. In contrast, external vascularized TH layer and interstitial stroma contain lower lipid storage compared to GC. This may be due to constant and intense transfer of lipids (in the form of HDLs or associated with albumin) from the TH cells [2], and a high activity of lipid consumption through FAO in TH cells, in which the correspondent enzymes were strongly expressed, as shown in porcine [36] and bovine [13]. Formation of lipid droplets occurs in the endoplasmic reticulum and they are potential metabolic units due to their localization close to mitochondria. Lipid droplets contain a hydrophobic core of neutral lipids, from TG and CE, surrounded by a monolayer of phospholipids and proteins as perilipins, calveolin and others [81], and these lipids are degraded by different lipases present on their surfaces. Overexpression of *PLIN2* in TH of small follicles indicates more lipid storage compared to LF. Furthermore, in our work, small and intermediate follicles (2–6 mm) showed the most intense accumulation of lipid droplets in mural GC and internal TH layers comparing to similar compartments of very small follicles (<1 mm) or large dominant follicles (>10 mm), where lipid droplets were distributed homogeneously through GC, internal and external TH layers.

Interestingly, spatial distribution of accumulated lipid droplets in GC and internal TH associated not only to follicle size but also to degree of follicular atresia. Indeed, ovarian follicular atresia is initiated with the apoptosis of GC [82]. Apoptotic GC expressed active caspase 3 in human [35] and bovine [83]. According to our data, GC from the follicles with higher atresia degree (caspase 3 positive) seem to accumulate less lipid droplets than from more healthy ones (caspase 3 negative). This result corroborates with the observation of lower accumulation of lipid droplets in hepatic cells ongoing apoptosis [84]. However, slight follicular atresia in GC of small follicles did not affect oocyte quality and even increased embryo development rate *in vitro* [23]. In human, lipid composition of healthy and apoptotic lung tissues significantly differed, showing an increase of pro-apoptotic ceramide species in affected tissue [85]. In addition, pharmacological inhibition of lipid droplets accumulation led to apoptosis of colon cancer cells [86]. Through the ovary, the follicles have different degree of cell apoptosis that may explain the variability of lipid content between the follicles of similar size, both in cellular TH and GC layers and in FF, as was here observed by MALDI MS lipid profiling.

Taken together, our data demonstrate that lipid composition of large dominant and small subordinate follicles significantly differed, especially in follicular fluid and granulosa/cumulus cells, whereas lipid content of enclosed immature oocyte was more stable. Hence, specific lipids from FF, GC and CC seem able to affect further oocyte competence; therefore, entering dominant follicle growth, the enclosed oocyte may serve accumulated lipids to support further ovulation and embryo development.

## 4. Materials and Methods

### 4.1. Ethics

No experiments on living animals were performed. Bovine ovaries were obtained from a local commercial slaughterhouse.

### 4.2. Chemicals

Unless indicated, all chemicals were purchased from Sigma-Aldrich (Saint-Quentin Fallavier, France).

### 4.3. Biological Materials

Bovine ovaries were transported from the commercial slaughterhouse on ice. Ovaries selected for carrying follicles both small and large follicles were either immediately frozen under nitrogen stream or used for follicle dissection. Frozen ovaries were kept at –80 °C and the ovarian crysections were made within 4 days after freezing and used immediately for Nile Red Fluorescence staining (NRF) of lipids or MALDI MS imaging (MSI) of lipids.

Dissection of the largest follicle and several smaller follicles more than 3 mm diameter were performed on freshly collected ovaries (*n* = 24). Individual follicles from the same ovary were isolated and measured with a caliper rule. Follicles (3–19 mm) were then dissected in order to separate FF, scraping off GC and TH cell layers. Briefly, each follicle was dissected on a glass Petri dish; interior GC were gently scrapped off from the interior walls of the follicles and aspirated with FF. FF from each follicle was clarified by centrifugation (10 min 5000× *g* at 4 °C), and GC pellets were then washed with PBS. Empty follicles were rigorously rinsed with PBS, and then a thin layer of internal TH was detached from each follicle. GC and TH samples were put into separate tubes with sterile cold phosphate buffered saline (PBS) and centrifuged (10 min 5000× *g* at 4 °C). Cell pellets were then washed twice in Tris-Sucrose (TRIS-S) buffer (260 mM sucrose 20 mM Tris-HCl pH 6.8; TRIS-S), centrifuged and most of the TRIS-S removed. Cell-free FFs and follicular cells from individual follicles were snap-frozen in liquid nitrogen.

Cumulus-oocyte complexes (COC) were aspirated from the small follicles (SF, 3–7 mm in diameter) and large follicles (LF, >8 mm in diameter) of 24 ovaries separately, using a 21-gauge needle attached to a 1 or 5 mL syringe, and collected fluids were allocated to independent tubes for each follicle size. COC with several CC layers were then selected. The individual oocytes were separated from their CC by repeating pipetting, and then rinsed twice in TRIS-S buffer. CC samples were washed in sterile PBS, centrifuged for 10 min 5000× *g* at 4 °C and the pellets were washed twice in TRIS-S. All the samples were snap-frozen in liquid nitrogen and kept at −80 °C to analyze within no longer than 4 days.

For RNA analysis, GC and TH cells from 12 individual follicles per group (SF and LF) were collected by centrifugation as described and kept in 200 μL of Trizol reagent (Invitrogen, Cergy Pontoise, France) at −80 °C until RNA extraction.

### 4.4. Mass Spectrometry Imaging by MALDI-TOF MS

Frozen ovaries (*n* = 7) stored at −80 °C were placed at −20 °C and cut at 10 μm thick sections using a Cryo-Star HM 560 cryostat (Microm, Francheville, France) at −18 °C. The sections were thaw-mounted onto conductive Indium Tin Oxide-coated microscope slides (Bruker Daltonics, Wissembourg, France) and placed under vacuum in a dessicator to encure complete drying. Using a histology slide scanner (OpticLab H850 scanner, Plustek, Ahrensburg, Germany) the ovary sections were scanned before matrix deposition to overlay the histological and molecular images. CHCA (α-cyano-4-hydroxycinnamic acid) matrix was freshly prepared at a concentration of 7 mg/mL in acetonitrile/H_2_O (60:40, *v*/*v*) with 0.2% trifluoroacetic acid. The tissue sections were coated with the CHCA using the ImagePrep sprayer device (Bruker Daltonik GmbH, Bremen, Germany), and then vacuum-dried during 1 h.

Spectra were acquired using an UltrafleXtreme MALDI-TOF instrument (Bruker Daltonik GmbH, Bremen, Germany) equipped with a Smartbeam laser (Nd:YAG, 355 nm) monitored by the FlexControl 3.4 software (Bruker Daltonics, Bremen, Germany). For each MSI sequence, the instrument was externally calibrated using a mixture of known small molecules and peptides. One μL of calibrant solution containing Cafein, MRFA peptide, Leu-Enkephalin, Bradykinine 2–9, Glu1-fibrinopeptide B; reserpine; Bradykinine; and Angiotensine I was mixed (1:1 *v*/*v*) with the CHCA matrix.

Each MSI sequence was performed from one ovarian section targeting one region of interest (ROI) corresponding to previously scanned ovary image.

Spectra were acquired in the positive mode, at a 2.0-kHz laser repetition rate, in the 100–1200 *m*/*z* range, using a spatial resolution set at 100 μm (medium focus setting) and collecting 500 spectra per pixel as a sum of 50 consecutive laser shots in 10 random walk shot steps. For mass accuracy across a tissue section image, internal calibration was applied to all of the spectra, using a lock mass set at *m*/*z* 760.5780 corresponding to [M + H]^+^ of PC 34:1. The unprocessed MSI sequence was then imported into the SCiLS Lab software (version 2016b, SCiLS, GmbH, Bremen, Germany). MSI sequence was loaded and pre-processed; data were baseline reduced using a convolution algorithm with the setting of 20 for peak width and normalized to the total ion count (TIC). For ROI, two-dimensional (2D) ion density maps using medium background were created from the average projection spectrum, and after data partitioning (bisecting k-Means), a segmentation map was generated.

### 4.5. MALDI-TOF MS Profiling of Lipids in Follicular Cells and Fluid

Lipid profiles were performed on individual frozen oocytes, FF and somatic cells from individual follicles, divided in two diameter groups: 12 small follicles (SF, 3–7 mm, mean size 5.0 ± 0.21 mm) and 12 large follicles (LF > 8.0 mm, mean size 12.9 ± 1.27 mm). The oocytes were thawed on ice and placed on the MTP Ground Steel 384 MALDI plate (Bruker Daltonics, Bremen, Germany) to dry. Other samples were defrosted on ice, and five microliters of cell suspension or FF were dried in SpeedVac System (SPD 1010-230, Thermo Savant Eletronic Corporation, France) for 10 min at 45 °C. 2,5-dihydroxyacetophenone matrix (DHAP) solution was prepared in concentration 20 mg/mL (DHAP matrix at solubilized in 90% methanol, 2% trifluoroacetic acid in water). Cell pellets and FF samples were re-suspended in 4 μL and 8 μL of DHAP matrix-solution, respectively, sonicated for four min in ultrasonic bath (FisherBrand 15,052, Fisher Scientific, Illkirch-Graffenstaden, France) to solubilize lipids. Matrix and sample were mixed (1:1 *v*/*v*) before deposition of 1 μL onto MALDI plate and 1.5 μL of the same DHAP matrix-solution was added. Samples were spotted in three replicates (with the exception of a single oocyte) and dried at room temperature for 30 min. External calibration was performed using a mixture of small molecules and peptides as described previously for MSI. One μL of calibrant solution was mixed with 1 μL of the DHAP matrix-solution. A total of 3000 spectra per spot (as a sum of 1000 consecutive laser shots in three shot steps) were acquired, using a Bruker UltrafleXtreme MALDI-TOF instrument (Bruker Daltonics, Bremen, Germany) operating in positive and negative reflectron ion modes, in the 100–1200 *m*/*z* range. An internal calibration was subsequently applied to all of the spectra with a lock mass correction performed on the highest intensity peak, corresponding to the glycerophosphatidylcholine (PC 32:1, *m*/*z* 732.556) in the positive mode, and glycerophosphatidylinositol (PI 38:4; *m*/*z* 885.5499) in the negative mode. Each spectrum was converted to atxt file using FlexAnalysis 3.4 software and processed using MALDI Progenesis software version 1.2 (Nonlinear Dynamics, Newcastle upon Tyne, UK). Automatically detected peaks were analyzed considering a signal on noise ratio of five, with exclusion of isotopes. The coefficient of variation (CV%) of intensity values (normalized peak height, NPH) was calculated for all peaks in three technical replicates per sample in twelve different follicles for each tissue (Results: Table 5). For comparative analysis of the lipid peaks, the NPH values were used. One-way (ANOVA) and Tukey’s post-hoc test were used for differential analysis using XLSTAT software (Addinsoft, Paris, France). The differences were considered significant with *p* ≤ 0.05 and fold change > 2.

### 4.6. Lipid Characterization

The annotation of *m*/*z* features was based on lipid identifications described earlier [13] and the LIPID MAPS database.

### 4.7. Gene Expression Analysis

#### 4.7.1. RNA Extraction and Reverse Transcription

Total RNA from GC and TH cells of small and large follicles (SF, from 3–6 mm, mean size 5.0 ± 0.24 mm; LF, >8 mm, mean size 12.8 ± 1.24 mm, respectively) from 12 individual ovaries were extracted using TriZol reagent (Invitrogen, Cergy Pontoise, France) and then treated by RQ1 DNAse (Promega, Charbonnières, France) following the manufacturer’s instructions. After isopropanol precipitation, the RNA concentration was determined using a NanoDrop ND-1000 spectrophotometer (Nyxor Biotech, Paris, France). Reverse transcription (RT) was performed on 200 ng of extracted RNA from somatic cells, using the Maxima First Strand cDNA Synthesis kit (Thermo-Fisher Scientific, Courtaboeuf, France) according to the manufacturer’s instructions.

#### 4.7.2. Real Time PCR Analysis

Real-time PCR reactions were carried out on a CFX96 (Bio-Rad, Marnes-la-Coquette, France) in 20 μL reaction mix containing primers at a final concentration of 150 nM each, 5 μL of the diluted RT reaction (equivalent of 2 ng converted RNA), and qPCR Mastermix Plus for SYBR Green I (Bio-Rad, Marnes-la-Coquette, France) according to the manufacturer’s instructions. The efficiency of the primers (Appendix A, Table A1) and standard curve for each gene were deduced from serial dilutions of the correspondent cDNA fragment as a template. The geometric mean of three housekeeping genes (*RPL19, RPS9* and *GAPDH*) was used to normalize gene expression. The relative expression of the genes of interest (RE) was calculated according to the equation: R = (E_gene^(-Ct gene))/(geometric mean (E_*RPS9*^(-Ct *RPS*9); E_*RPL1*9^(-Ct *RPL19*); E_*GAPD*H^(-Ct *GAPDH*))), where E is the primer efficiency and Ct the cycle threshold. Statistical analysis was made by Mann Whitney test using GraphPad Prism version 5.01 for Windows, (GraphPad Software, La Jolla California USA, www.graphpad.com). Difference was considered significant at *p* < 0.05.

### 4.8. Isolation of Extracellular Vesicles from Follicular Fluid

Extracellular vesicles (EVs) were isolated from FF aspirated from SF (3–6 mm, 6 pools of 1–3 mL FF) and LF (>8 mm, 6 pools of 1–3 mL FF) by serial ultracentrifugation at 4 °C. First, FFs were centrifuged at 300× *g* 15 min to eliminate cells, than supernatant FF were centrifugated at 2000× *g* 15 min at room temperature to eliminate cell debris and apoptotic bodies. Then clarified FFs were centrifuged 30 min at 12000× *g* at 4 °C. Pellets containing the largest EVs (usually named microvesicles, MVs) were rinsed with PBS, centrifuged 5 min and resuspended in 20 μL of PBS per 1 mL of FF. Supernatant FF were ultracentrifuged at 100,000× *g* for 90 min at 4 °C (Beckman model L8-M with SW-55-Ti rotor, adjusted k-factor: 163, using the formula k = (2.533 10^11^) × ln(rmax/rmin)/RPM^2^, were r(min) = 60.8 mm and r(max) = 108.5 mm at 30,000 RPM). Pellets were diluted with 4 mL of PBS and ultracentrifuged again at 100,000× *g* for 90 min at 4 °C. The final pellet, containing the smaller EVs (exosome–like nanovesicles), was resuspended with 25 μL of PBS per one mL of original FF. EVs suspensions (2.5 μL) were fixed in 5 μL of 2% glutaraldehyde solution in PBS. Vesicle abundance was estimated by protein quantification of 10-fold dilutions of EVs preparations in water (2 μL of EV suspension with 18 μL of distilled water) using Bicinchoninic acid Assay (BCA; Interchim, Montluçon, France) according to manufacturer’s instructions using BSA as a standard.

### 4.9. Transmission Electron Microscopy (TEM)

TEM was used to characterize EVs isolated from FF (ffEV) of SF and LF. Transmission electron microscopy analysis was performed on six independent samples per class using the aliquots from each ffEV suspensions described above. A volume of 3 μL of fixed ffEVs was placed on formvar and carbon-coated grid and incubated for 5 min at room temperature. Samples were washed with distilled water trice, then stained with 2% uranyl acetate and air dried at room temperature. The micrographs were obtained using TEM HITACHI HT 7700 Elexience at 80 kV (with a charge-coupled device camera AMT) and JEM 1011 (JEOL, Tokyo, Japan) equipped with a Gatan digital camera driven by Digital Micrograph software (Gatan, Pleasanton, CA, USA) at 100 kV. The images were used to measure EVs size with ImageJ software (NIH, Bethesda, MD, USA). Comparison of microvesicle and exosome size distribution was carried out using Chi2-test by XLSTAT (Addinsoft, Paris, France).

### 4.10. Nile Red Fluorescence (NRF)

Nile Red staining in bovine ovary sections was mainly performed as previously described [13]. Briefly, frozen bovine ovaries (*n* = 7) were cut in 10-μm sections using cryostat (MICROM NX70, Walldorf, Germany) at −18 °C. Frozen sections were mounted onto Superfrost ^®^Plus glass slides (Thermo Scientific, Villebon sur Yvette, France) and air dried for 10 min. Sections were fixed in 4% (*v*/*v*) of paraformaldehyde during 2 min, and then washed in water during 5 min at room temperature. The slides were incubated in 500 ng/μl Nile red water solution for 1 h at room temperature, after washed 5 min in water and mounted in Moveol solution complemented with 1 μg/mL of 4′,6-Diamidine-2′-phenylindole dihydrochloride (DAPI).

The slides were scanned using AxioScan.Z1 scanner (Carl Zeiss SAS, Oberkochen, German) at 20×/0.8 magnification. Specific filters were used to detect blue DAPI fluorescence (excitation and emission wavelength were 335–383 nm and 420–470 nm, respectively) and Nile Red Fluorescence (excitation 538–562 nm and emission 570–640 nm). The exposure time used for all the slides were 50 ms to DAPI and 35 ms to NRF. Each image was analyzed using Zen Blue version 6.1.7601 software (Carl Zeiss Microscopy GmbH, Oberkochen, German). The intensity of fluorescence of NRF and DAPI were measured in in regions sufficient to cover the entire compartment of each follicle, named regions of interest (ROIs), located to external theca (eTH), internal theca (iTH) and granulosa cell (GC) layers, representing about a 2 mm^2^ ROI per compartment and per individual follicle. Fluorescence background apart of the cells was subtracted from DAPI and NRF intensity values, respectively, and resulted value was then normalized by cells number in each ROI. For statistical analysis, 43 follicles from 13 ovaries were grouped by size, defined from 12 images per ovary. Comparison of normalized NRF intensities between GC, iTH and eTH of different groups were performed by one-way ANOVA followed Newman-Keuls multiple comparison test using GraphPad Prism version 5.01 for Windows, (GraphPad Software, La Jolla, CA, USA, www.graphpad.com).

### 4.11. Immunohistochemistry on Ovary Sections

Sections of frozen bovine ovaries measuring 10 μm were fixed in 4% paraformaldehyde buffered PBS during 2 min. The slides were rinsed once in TBS (Tris-buffered saline, 10 mM Tris, 150 mM NaCl, ph 7.4) for 5 min, and then blocked with horse serum blocking solution for 20 min. The slides were incubated overnight at 4 °C with the primary antibodies against Caspase-3 (CASP3, 1:500, rabbit polyclonal, Cusabio, Wuhan Huamei Biotech Co., Hubei Province, China, CSB-PA05689A0Rb) in TBST (TBS with 0.1% Triton ×100) with 5% non-fat dry milk. In control slides, rabbit IgG replaced primary antibody. Slides were washed three times in TBS (5 min), and incubated 2 h at room temperature with secondary anti-rabbit biotinylated antibody (1:2000, Biotinylated Universal Antibody, Horse Anti-Mouse/Rabbit IgG, Vector, BA-1400) and rinsed in TBS (5 min). The signal revelation was made with VECTASTAIN ABC-AP Staining Kit (Vector, AK-5000) as recommended by the manufacturer. The sections were counterstained with Papanicolaou blue stain. Coverslips were applied with aqueous mounting medium after dehydration through graded alcohol baths and toluene (1 min). Sections were examined using an Axioplan Carl Zeiss microscope. Images were acquired using a digital monochrome camera (Spot-Flex, Diagnostic Instruments, Michigan, USA) coupled with the SPOT 5.2 imaging software. Images were analyzed by ImageJ version 1.51n (http://imagej.nih.gov/ij, NIH, USA).

## 5. Conclusions

In the present study, we have demonstrated that lipid composition significantly changed during follicle terminal growth in both follicular fluid and somatic cells, especially in granulosa and cumulus cells, with mitigated modulation of lipid composition of the enclosed immature oocyte. Follicular compartment-dependent turnover and accumulation of specific lipid species, involved in maintenance of plasma membrane integrity, energy storage and signaling, could affect intra-follicular communication during final follicular growth and might be involved in progress to either follicular atresia or dominance. Lipids in large dominant follicles contained more long-chain and very long chain FAs, which are poorly metabolized by mitochondria to immediate energy and which could be stored for future use, as post-ovulation progesterone production by corpus luteum. Theca and granulosa cell layers might regulate intra-follicular lipid metabolism through FA uptake and transformations, thus leading to strong modulation of FF lipid composition, which related to follicle size and atresia degree. Among the follicular compartments, lipid composition of FF is the most variable likely due to differences in extracellular vesicle composition and secreting activities of follicular cells. In part due to significant lipid modulations in surrounding cumulus cells, lipid composition of enclosed immature oocyte seems being highly regulated, and accumulated lipids might serve to provide energy, signaling molecules and building blocks to support further ovulation and early embryo development.

## Figures and Tables

**Table 1:** Detection and differential analysis of lipids * in follicular cells and fluids between the large and small follicles.

Follicular Compartment	Number of Detected Peaks	Differentially Abundant Peaks, *n* (%)	LF vs. SF (Relative Abundance of the Peaks)	
			Up-Regulated (*n*)	Down-Regulated (*n*)
OO	468	8 (1.7%)	2	6
CC	439	43 (9.8%)	41	2
FF	375	208 (55.5%)	111	97
GC	350	52 (14.9%)	35	17
TH	338	18 (5.3%)	15	3

* Lipids detected in positive and negative modes in the range 300–1000 *m*/*z* were compared between the LF and SF (*p* < 0.05; fold change >2). Abbreviations: LF—large follicle; SF—small follicle.

**Table 2:** Annotated differential lipids in follicular fluid of large and small follicles.

*m*/*z*	*p*-Value	Ratio LF vs. SF	Lipid Ion (Carbons: Unsaturation [Add])
*m/z* 546.293^+^	5.84 × 10^7^	6.14	LPC 18:0 [Na]^+^
*m/z* 524.310^+^	6.21 × 10^7^	5.21	LPE 20:4 [Na]^+^ or LPC 18:0 [H]^+^
*m/z* 671.543^+^	7.21 × 10^5^	3.99	CE 18:2 [Na]^+^
*m/z* 506.197^+^	1.33 × 10^6^	3.90	LPC 14:0 [K]^+^
*m/z* 858.613^+^	1.02 × 10^4^	3.49	PC 42:8 [H]^+^ or PC 40:5 [Na]^+^
*m/z* 860.595^+^	7.80 × 10^5^	3.31	PC 42:7 [H]^+^ or PC 40:4 [Na]^+^
*m/z* 810.601^+^	2.75 × 10^8^	2.82	PC 38:4 [H]^+^
*m/z* 808.592^+^	2.73 × 10^5^	2.74	PC 38:5 [H]^+^
*m/z* 518.223^+^	2.88 × 10^4^	2.56	LPC 18:3 [H]^+^
*m/z* 544.283^+^	4.55 × 10^6^	2.46	LPC 18:1 [Na]^+^
*m/z* 788.619^+^	6.14 × 10^6^	2.43	PC 36:1 [H]^+^
*m/z* 832.584^+^	1.47 × 10^6^	2.39	PC 40:7 [H]^+^ or PC 38:4 [Na]^+^
*m/z* 812.625^+^	2.32 × 10^7^	2.37	PC 38:3 [H]^+^
*m/z* 711.516^+^	1.80 × 10^4^	2.36	SM 33:1 [Na]^+^
*m/z* 669.576^+^	5.15 × 10^5^	2.34	CE 18:3 [Na]^+^
*m/z* 790.629^+^	2.34 × 10^5^	2.07	PC O-38:7 or PC P-38:6 [H]^+^
*m/z* 716.424^+^	1.87 × 10^4^	2.01	PC 28:0 [K]^+^
*m/z* 872.530^+^	1.44 × 10^2^	0.49	PC 40:6 [K]^+^
*m/z* 605.487^+^	3.98 × 10^5^	0.48	DG 32:1 [K]^+^
*m/z* 683.450^+^	1.44 × 10^3^	0.48	DG 38:4 [K]^+^
*m/z* 502.218^+^	3.15 × 10^3^	0.46	LPC P-16:0 [Na]^+^
*m/z* 826.575^+^	2.98 × 10^2^	0.46	PC 36:1 [K]^+^
*m/z* 748.537^+^	6.20 × 10^4^	0.43	PC 33:0 [H]^+^
*m/z* 834.607^−^	9.57 × 10^7^	0.43	PS 40:6 [H]^−^
*m/z* 846.501^+^	2.10 × 10^2^	0.41	PC 38:5 [K]^+^
*m/z* 870.506^+^	7.08 × 10^11^	0.41	PC 40:7 [K]^+^
*m/z* 794.668^−^	1.21 × 10^9^	0.38	PE 40:4 [H]^−^
*m/z* 800.540^+^	7.12 × 10^10^	0.37	PC 34:0 [K]^+^
*m/z* 706.527^+^	1.20 × 10^7^	0.33	PC 30:0 [H]^+^
*m/z* 770.517^+^	1.12 × 10^12^	0.32	PC 32:1 [K]^+^
*m/z* 762.576^+^	3.23 × 10^10^	0.31	PC 34:0 [H]^+^
*m/z* 741.510^+^	5.38 × 10^10^	0.30	SM 34:1 [K]^+^
*m/z* 769.515^+^	8.65 × 10^9^	0.30	SM 36:1 [K]^+^
*m/z* 887.662^−^	1.23 × 10^7^	0.30	PI 38:3 [H]^−^
*m/z* 707.525^+^	2.32 × 10^7^	0.29	DG 40:6 [K]^+^
*m/z* 716.652^−^	3.02 × 10^6^	0.27	PE 34:1 [H]^−^
*m/z* 750.654^−^	6.63 × 10^6^	0.27	PE O-38:5 or PE P-38:4 [H]^−^
*m/z* 798.517^+^	1.51 × 10^11^	0.23	PC 34:1 [K]^+^
*m/z* 846.658^−^	2.45 × 10^11^	0.23	PS P-42:6 [H]^−^
*m/z* 772.501^+^	4.55 × 10^13^	0.21	PC 32:0 [K]^+^
*m/z* 734.551^+^	3.02 × 10^12^	0.16	PC 32:0 [H]^+^
*m/z* 774.645^−^	1.45 × 10^10^	0.14	PE O-40:7 or PE P-40:6 [H]^−^
*m/z* 885.652^−^	9.09 × 10^10^	0.11	PI 38:4 [H]^−^
*m/z* 742.641^−^	1.97 × 10^8^	0.10	PE 36:2 [H]^−^
*m/z* 760.606^−^	2.60 × 10^5^	0.10	PE 38:7 [H]^−^
*m/z* 836.624^−^	4.67 × 10^8^	0.10	PS 40:5 [H]^−^
*m/z* 883.624^−^	2.32 × 10^8^	0.09	PI 38:5 [H]^−^
*m/z* 744.664^−^	8.79 × 10^12^	0.07	PE 36:1 [H]^−^
*m/z* 788.660^−^	1.92 × 10^9^	0.02	PS 36:1 [H]^−^

**Table 3:** Annotated differential lipids in oocytes of large and small follicles.

*m*/*z*	*p*-Value	Ratio LF vs. SF	Lipid Ion (Carbons: Unsaturation [Add])
*m/z* 702.431^+^	3.66 × 10^2^	0.43	PC O-31:2 or PC P-31:1 [H]^+^
*m/z* 728.456^+^	3.41 × 10^2^	0.43	PC 29:1 [K]^+^
*m/z* 713.467^+^	2.28 × 10^2^	0.39	SM 32:1 [K]^+^

**Table 4:** Annotated differential lipids in cumulus cells of large and small follicles.

*m*/*z*	*p*-Value	Ratio LF vs. SF	Lipid Ion (Carbons: Unsaturation [Add])
*m/z* 714.352^+^	2.93 × 10^5^	4.65	PC 29:0 [Na]^+^
*m/z* 734.595^+^	7.08 × 10^3^	2.90	PC 32:0 [H]^+^
*m/z* 813.701^+^	8.74 × 10^5^	2.78	TG 47:1 [Na]^+^
*m/z* 808.598^+^	4.51 × 10^4^	2.57	PC 38:5 [H]^+^
*m/z* 828.758^+^	2.28 × 10^3^	2.57	PC 39:2 [H]^+^
*m/z* 800.647^+^	7.75 × 10^3^	2.49	PC 34:0 [K]^+^ or PC 37:2 [H]^+^
*m/z* 810.634^+^	2.96 × 10^4^	2.45	PC 38:4 [H]^+^
*m/z* 824.744^+^	3.46 × 10^5^	2.45	PC O-40:4 or PC P-40:3 [H]^+^
*m/z* 834.642^+^	2.54 × 10^5^	2.35	PC 40:6 [H]^+^ or PC 38:3 [Na]^+^
*m/z* 826.754^+^	7.79 × 10^3^	2.34	PC 39:3[H]^+^
*m/z* 786.626^+^	1.97 × 10^3^	2.33	PC 36:2 [H]^+^ or PC 33:0 [K]^+^
*m/z* 836.677^+^	3.26 × 10^4^	2.13	PC 40:5 [H]^+^ or PC 38:2 [Na]^+^
*m/z* 725.533^+^	9.83 × 10^3^	2.06	SM 34:1 [Na]^+^
*m/z* 748.593^+^	6.23 × 10^5^	2.06	PC 33:0 [H]^+^
*m/z* 788.634^+^	1.28 × 10^3^	2.06	PC 36:1 [H]^+^
*m/z* 802.659^+^	3.46 × 10^2^	2.02	PC 37:1 [H]^+^
*m/z* 744.522^+^	1.57 × 10^3^	2.01	PC O-31:0 [K]^+^
*m/z* 721.406^+^	2.38 × 10^2^	2.00	SM 34:3 [Na]^+^

**Table 5:** Annotated differential lipids in granulosa cells of large and small follicles.

*m*/*z*	*p*-Value	Ratio LF vs. SF	Lipid Ion (Carbons: Unsaturation [Add])
*m/z* 861.431^−^	5.83 × 10^3^	2.81	PI 36:2 [H]^−^
*m/z* 844.546^+^	1.03 × 10^4^	0.50	PC 38:6 [K]^+^
*m/z* 560.274^+^	3.70 × 10^3^	0.49	LPC 18:1 [K]^+^
*m/z* 863.704^+^	2.54 × 10^2^	0.47	SM 44:2 [Na]^+^
*m/z* 848.559^+^	1.01 × 10^4^	0.46	PC 38:4 [K]^+^
*m/z* 722.463^−^	8.16 × 10^4^	0.45	PE O-36:5 or PE P-36:4 [H]^−^
*m/z* 582.303^+^	1.22 × 10^2^	0.40	LPC 20:4 [K]^+^
*m/z* 534.302^+^	3.84 × 10^3^	0.38	LPC 16:0 [K]^+^
*m/z* 428.379^+^	8.21 × 10^4^	0.37	C18:0 Carnitine [H]^+^

**Table 6:** Annotated differential lipids in theca cells of large and small follicles.

*m*/*z*	*p*-Value	Ratio LF vs. SF	Lipid Ion (Carbons: Unsaturation [Add])
*m/z* 667.572^+^	2.20	2.2	DG 38:4 [Na]^+^
*m/z* 683.481^+^	2.14	2.14	DG 38:4 [K]^+^
*m/z* 760.544^−^	2.08	2.08	PE 38:7 [H]^−^
*m/z* 714.550^−^	2.02	2.02	PE 34:2 [H]^−^

**Table 7:** Gene expression analysis in theca and granulosa cells from small and large follicles.

	Theca Cells		Granulosa Cells	
Gene	Small Follicles	Large Follicles	Small Follicles	Large Follicles
*ACACA*	1579.6 ± 373.7	1044.4 ± 246.6	986.4 ± 309.1	906.6 ± 325.8
*ACADVL*	1463.2 ± 674.1	4280.2 ± 3191.1	*1692.7 ± 816.9*	*4716.8 ± 1635.5*
*ACOT9*	**11.8 ± 2.9**	**8.1 ± 3.5**	0.88 ± 0.2	1.04 ± 0.2
*APOA1*	0.42 ± 0.2	0.31 ± 0.1	0.22 ± 0.04	0.42 ± 0.2
*CD36*	*253.3 ± 69.5*	*168.9 ± 89.6*	2302.1 ± 979.9	1820.9 ± 778.8
*CPT2*	12774.0 ± 4910.7	10332.6 ± 5231.8	8687.4 ± 2408.4	8464.22 ± 3618.4
*CYP11*	1740.53 ± 1205.2	5463.51 ± 2995.6	**1583.9 ± 547.3**	**9277.1 ± 3107.9**
*FABP3*	*3346.1 ± 1456.9*	*1573.1 ± 298.9*	859.8 ± 241.7	1629.3 ± 540.7
*FABP5*	*6860.6 ± 2342.1*	*8872.8 ± 4077.2*	547.9 ± 272.5	1646.8 ± 1043.2
*GLUT1*	965.2 ± 255.6	3198.5 ± 2068.3	13126.3 ± 5844.3	44136.2 ± 23953.6
*GPX4*	2927.1 ± 1100.8	12189.2 ± 7173.6	*11672.5 ± 3132.3*	*34370.6 ± 8799.6*
*HADHA*	852.1 ± 236.7	883.7 ± 156.9	885.1 ± 137.7	1229.3 ± 130.3
*HSD3B1*	**1060.6 ± 331.9**	**566.9 ± 189.5**	885.3 ± 291.9	1387.5 ± 330.5
*LPL*	*164762.8 ± 57444.5*	*149005.3 ± 77915.5*	973.7 ± 388.5	1055.4 ± 424.3
*PLIN2*	**18.5 ± 6.5**	**12.4 ± 6.5**	5.9 ± 2.6	2.7 ± 0.6
*SCARB1*	10.9 ± 4.3	15.7 ± 4.6	16.3 ± 8.1	18.6 ± 5.0
*SCARB2*	2.7 ± 2.0	3.1 ± 1.4	7.7 ± 3.2	11.5 ± 4.7
*TRIB2*	**5.76 ± 1.4**	**6.26 ± 3.3**	1.92 ± 0.9	3.96 ± 1.9

Mean ± SD values of 12 samples per group are shown. Significantly different values are in bold (*p* < 0.05). Tendency to significance is marked in italics (*p* < 0.1). *ACACA*—Acetyl-CoA Carboxylase Alpha; *ACADVL*—Acyl CoA Dehydrogenase Very Long Chain; *ACOT9*—Acyl-CoA Thioesterase 9; *APOA1*—Apolipoprotein A1; *CD36*—CD36 Molecule (Thrombospondin Receptor, Fatty Acid Translocase); *CPT2*—Carnitine Palmitoyltranferase 2; *CYP11A1*—Cytochrome P450 Family 11 Subfamily A Member 1; *FABP3*—Fatty acid binding protein 3; *FABP5*—Fatty acid binding protein 5; *GLUT1*—Glucose transporter 1; *GPX4*—Glutathione peroxidase 4; *HADHA*—Hydroxyacyl-CoA Dehydrogenase Trifunctional Multienzyme Complex Subunit α; *HSD3B1*—Hydroxy-Delta-5- Steroid Dehydrogenase, 3 Beta- and Steroid Delta-Isomerase 1; *LPL*—Lipoprotein Lipase; *PLIN2*—Perilipin 2; *SCARB1*—Scavenger Receptor Class B Member 1; *SCARB2*—Scavenger Receptor Class B Member 2; *TRIB2*—Tribbles homolog 2.

## Supplementary Materials

The following are available online at https://www.mdpi.com/1422-0067/21/18/6661/s1.

## Abbreviations

ACACA	Acetyl-CoA Carboxylase Alpha
ACADVL	Acyl-CoA Dehydrogenase Very Long Chain
ACOT9	Acyl-CoA Thioesterase 9
APOA1	Apolipoprotein A1
ATT	6-Aza-2-thiothymine matrix
Casp3	Caspase-3
cAMP	Cyclic adenosine monophosphate
CC	Cumulus cells
CD36	CD36 Molecule (Thrombospondin Receptor, Fatty Acid Translocase)
Cer	Ceramide
CE	Cholesteryl esters
CHCA	α-Cyano-4-hydroxycinnamic acid matrix
CPT2	Carnitine Palmitoyltranferase 2
CYP11A1	Cytochrome P450 Family 11 Subfamily A Member 1
DAPI	4′,6-Diamidine-2′-phenylindole dihydrochloride
DG	Diacylglycerol
DHAP	2,5-dihydroxyacetophenone matrix
DHB	2,5-Dihydroxybenzoic acid matrix
EV	Extracellular vesicle
FA	Fatty acyl
FABP3	Fatty acid binding protein 3
FABP5	Fatty acid binding protein 5
FAO	Fatty acid oxidation
FF	Follicular fluid
ffEVs	Extracellular vesicles isolated from FF
GC	Granulosa cells
GLUT1	Glucose transporter 1
GPX4	Glutathione peroxidase 4
HABA	2-(4′-Hydroxybenzeneazo) benzoic acid matrix
HADHA	Hydroxyacyl-CoA Dehydrogenase Trifunctional Multienzyme Complex Subunit α
HDL	High Density Lipoprotein
HSD3B1	Hydroxy-Delta-5-Steroid Dehydrogenase, 3 Beta- and Steroid Delta-Isomerase 1
LF	Large Follicle
LPC	Lyso-phosphatidylcholines
LPE	Lyso-phosphatidylethanolamines
LPL	Lipoprotein Lipase
*m*/*z*	Ion Mass to Ion Charge number ratio
MALDI-TOF	Matrix Assisted Laser Desorption Ionization -Time of Flight
MBT	2-Mercaptobenzothiazole matrix
MS	Mass Spectrometry
MSI	Mass Spectrometry Imaging
MV	Microvesicle
NRF	Nile Red Fluorescence
OO	Oocyte
COC	Cumulus-oocyte complex
PC	Phosphatidylcholines
PE	Phosphatidylethanolamines
PI	Phosphatidylinositols
PI3K	Phosphatidylinositol-3-Kinase
PLIN2	Perilipin 2
PON1	Paraxoxonase 1
PS	Phosphatidylserines
ROI	Region of Interest
SCARB1	Scavenger Receptor Class B Member 1
SCARB2	Scavenger Receptor Class B Member 2
SCD	Stearoyl-CoA desaturase
SF	Small Follicles
SM	Sphingomyelins
SOD	Superoxide dismutase
TG	Triacylglycerol
TH	Theca cells
eTH	External Theca
iTH	Internal Theca
TRIB2	Tribbles homolog 2

## Appendix A

**Table A1:** List of primers used for RT-qPCR analysis of gene expression in bovine ovarian cells.

Gene	Accession Number	Description		Primer’s Sequence (5′-3′)	Efficiency% (E)
*ACACA*	NM_174224	Acetyl-CoA Carboxylase Alpha	*Fw* *Rev*	TGCTTCCCATTTGCCATC CTGCCATCCTCACGACCT	103.2% (2.03)
*ACADVL*	MN_174494	Acyl-CoA Dehydrogenase, Very Long Chain	*Fw* *Rev*	CATCGTCCACCAGGAACTGAG GAACCACCACCATGGCATAGA	103.129% (2.03)
*ACOT9*	NM_001034560.1	Acyl-CoA Thioesterase 9	*Fw* *Rev*	GTGAGGTGGCCTCTCTTCAG GGGCTTCAACAAGAGCAGTC	114.5% (2.14)
*APOA1*	NM_174242.3	Apolipoprotein A1	*Fw* *Rev*	TTTGGGAAAACAGCTCAACC CAGGTCCTTGTGCATCTCCT	84.1% (1.84)
*CD36*	NM_001044622.1	CD36-molecule (Thrombospondin Receptor)	*Fw* *Rev*	TTGGCCTATGAACCGTTTACT CGTTCTGAAGTTGCCAAGCA	119.6% (2.20)
*CPT2*	NC_037330.1	Carnitine Palmitoyltransferase 2	*Fw* *Rev*	AACCGCAAGTCTGGAGAAGA TGACCGTAACCACCATGAGA	105.1% (2.05)
*CYP11A1*	NM_176644.1	Cytochrome P450, Family 11, Subfamily A, Polypeptide 1	*Fw* *Rev*	CGGAAAGTTTGTAGGGGACA ACGTTGAGCAGAGGGACACT	100.5% (2.00)
*FABP3*	NM_174313	Fatty acid binding protein 3	*Fw* *Rev*	ATCGTGACGCTGGATGGCGG GCCGAGTCCAGGAGTAGCCCA	99.8% (1.99)
*FABP5*	NM_001129805.1	Fatty acid binding protein 5	*Fw* *Rev*	ACGTGTCGAAAGAAGGAGGT ACATCCCAAAGAGCATATCGTA	100.7% (2.00)
*GAPDH*	NM_001034034	Glyceraldehyde 3 phosphate dehydrogenase	*Fw* *Rev*	TTCAACGGCACAGTCAAGG ACATACTCAGCACCAGCATCAC	100.1% (2.00)
*GPX4*	NM_174770	Glutathione Peroxidase 4	*Fw* *Rev*	CGATACGCCGAGTGTGGTTTAC ACAGCCGTTCTTGTCAATGAGG	101% (2.01)
*HADHA*	NM_174335	Hydroxyacyl-CoA Dehydrogenase Trifunctional Multienzyme Complex Subunit Alpha	*Fw* *Rev*	CTGTACGGGGCACAGAAAAT TGCCAGAGTTCGTCTTCCTT	102.2% (2.02)
*HSD3B1*	NM_174343	Hydroxy-Delta-5-Steroid Dehydrogenase, 3 Beta- and Steroid Delta-Isomerase 1	*Fw* *Rev*	TCCACACCAGCACCATAGAA AAGGTGCCACCATTTTTCAG	102.2% (2.02)
*LPL*	NM_001075120	Lipoprotein lipase	*Fw* *Rev*	GGGTTTTGAGCAAGGGTACA GCCACAATGACCTTTCCAGT	111.1% (2.11)
*PLIN2*	NM_173980	Perilipin 2	*Fw* *Rev*	ACAACACACCCCTCAACTGG CTGCCTGCCTACTTCAGACC	95.0% (1.95)
*RPL19*	BC102223	Ribosomal protein L19	*Fw* *Rev*	AATCGCCAATGCCAACTC CCCTTTCGCTTACCTATACC	101.0% (2.01)
*RPS9*	BC148016	Ribosomial protein S9	*Fw* *Rev*	GGAGACCCTTCGAGAAGTCC GGGCATTACCTTCGAACAGA	95.8% (1.96)
*SCARB1*	NM_174597.2	Scavenger Receptor Class B, member 1 or CD36 Antigen-Like	*Fw* *Rev*	ATGCCCAGTATGTGCTCCTT AAGAAGCGGGGTGTAGGG	100.1% (2.00)
*SCARB2*	NM_001102153.1	Scavenger Receptor Class B, member 2	*Fw* *Rev*	TCCAGGCTTCTCCCACTAGA TTCTGGGCAGCATTCTCTTT	109.6% (2.10)
*SLC2A1 (GLUT1)*	NM_174602	Solute Carrier Family 2 Member 1 (facilitative glucose transporter)	*Fw* *Rev*	CTGATCCTGGGTCGCTTCAT ACGTACATGGGCACAAAACCA	100% (2.00)
*TRIB2*	NM_178317.3	Tribbles homolog 2 (Drosophila)	*Fw* *Rev*	GTGGCATGTAGTGCAGACC ACAGGACAAAGCACCAGAG	100.2% (2.00)
